# *N*-Phenyl-*N*-[(*E*)-2-(4,4,5,5-tetra­methyl-1,3,2-dioxaborolan-2-yl)ethen­yl]aniline

**DOI:** 10.1107/S2414314622000839

**Published:** 2022-01-28

**Authors:** Yuki Hatayama, Kazuto Akagi, Tsunehisa Okuno

**Affiliations:** a Department of Systems Engineering, Wakayama University, Sakaedani, Wakayama, 640-8510, Japan; Sunway University, Malaysia

**Keywords:** crystal structure, dioxaborolan-2-yl, resonance

## Abstract

The title compound, C_20_H_24_BNO_2_, has a polarized π-system due to significant resonance between the N—C(H)=C(H)—B and ionic N^+^=C(H)—C(H)=B^−^ canonical forms. The dihedral angles between the NC_2_B plane (r.m.s. deviation 0.0223 Å) and the C_3_N (r.m.s. deviation 0.0025 Å) and BCO_2_ (r.m.s. deviation 0.0044 Å) planes are 2.51 (12) and 3.09 (19)°, respectively. This indicates the lone pair of the nitro­gen atom and a vacant *p* orbital of the boron atom are conjugated with the central C=C bond. In comparison with the carbazole analogue [Hatayama & Okuno (2012 ▸). *Acta Cryst.* E**68**, o84], the C—N and C—B bonds are shorter. The results are well explained by the increase in the contribution of the N^+^=C(H)—C(H)=B^−^ canonical form in the title compound.

## Abstract 2

The title compound features a polarized π-system due to resonance between the N—C(H)=C(H)—B and ionic N^+^=C(H)—C(H)=B^−^ canonical forms.

## Graphical abstract

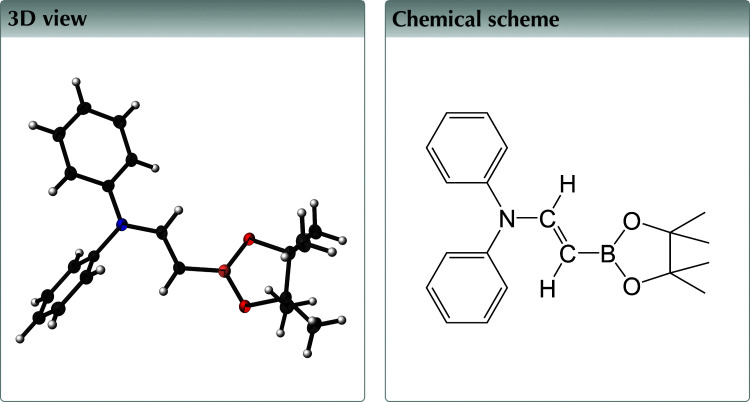

## Structure description

**Figure 1:**
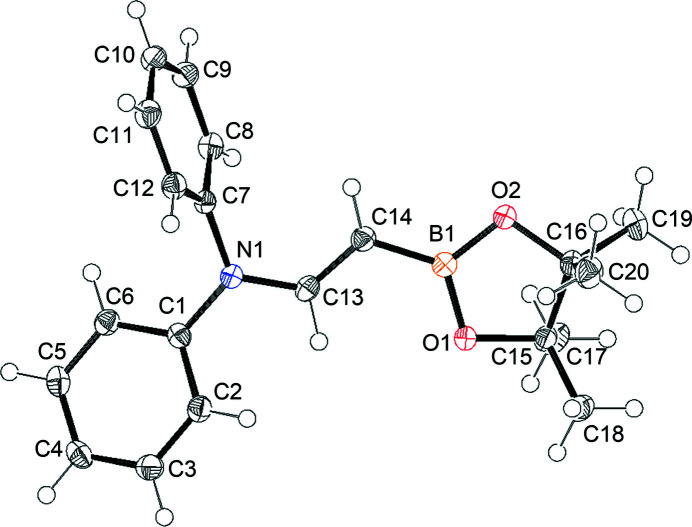
The mol­ecular structure of the title compound with displacement ellipsoids drawn at the 50% probability level; H are atoms shown as small spheres.

The title compound, C_20_H_24_BNO_2_, has a hybrid π-conjugated system within the N—C(H)=C(H)—B fragment. The insertion of a π-conjugated system in the N—B bond affords a highly polarized π-system owing to the contribution of an ionic canonical structure, *i.e*. N^+^= C(H)—C(H)=B^−^. The contribution of the ionic canonical structure is small when *p*-phenyl­ene is inserted into the N—B bond (Yuan *et al.*, 2006 ▸). However, when a C≡C bond is inserted into the N—B bond (Onuma *et al.*, 2015 ▸), a relatively large contribution of the ionic canonical structure is apparent. The structure of the C=C bond inserted system, namely 9-[(*E*)-2-(4,4,5,5-tetra­methyl-1,3,2-dioxaborolan-2-yl)ethen­yl]-9*H*-carbazole has been reported (Hatayama & Okuno, 2012 ▸). In the title compound, the carbazole unit of the former is replaced by a di­phenyl­amino residue (Fig. 1 ▸).

The dihedral angles between the C13/C14/B1/N1 plane (r.m.s. deviation 0.0223 Å) and the N1/C1/C7/C13 (r.m.s. deviation 0.0025 Å) and B1/O1/O2/C14 (r.m.s. deviation 0.0044 Å) planes are 2.51 (12) and 3.09 (19)°, respectively, indicating the lone pair of the nitro­gen atom and a vacant *p* orbital of the boron are conjugated with the central C=C bond. The C13—N1 [1.3824 (19) Å] and C14—B1 [1.532 (2) Å] bonds are shortened, compared with those in the carbazole analogue of 1.396 (3) Å and 1.537 (3) Å, respectively; the central C=C bond at 1.341 (2) Å is experimentally equivalent to that of 1.336 (4) Å in the carbazolyl derivative. The results are well explained by the increase in the contribution of the N^+^=C(H)—C(H)=B^−^ canonical structure in the title compound. This is presumably because the nitro­gen atom of di­phenyl­amino group donates its lone pair to the π-system more effectively compared to that of the carbazolyl group, which leads to a decrease in the contribution of the N^+^=C(H)—C(H)=B^−^ canonical structure in the latter.

## Synthesis and crystallization

The title compound was obtained by hydro­boration of *N*-ethynyl-*N*-phenyl­aniline (Tokutome & Okuno, 2013 ▸) with 4,4,5,5-tetra­methyl-1,3,2-dioxaborolane in 16% yield. ^1^H NMR (CDCl_3_): δ 1.25 (*s*, 12H), 4.17 (*d*, *J* = 15.6 Hz, 1H), 7.07 (*d*, *J* = 7.7 Hz, 4H), 7.12 (*t*, *J* = 7.7 Hz, 2H), 7.31 (*t*, *J* = 7.7 Hz, 4H), 7.64 (*d*, *J* = 15.6 Hz, 1H).

Single crystals were obtained by recrystallization from hexane solution.

## Refinement

Crystal data, data collection and structure refinement details are summarized in Table 1 ▸.

## Supplementary Material

Crystal structure: contains datablock(s) global, I. DOI: 10.1107/S2414314622000839/tk4073sup1.cif

Structure factors: contains datablock(s) I. DOI: 10.1107/S2414314622000839/tk4073Isup2.hkl

Supporting information file. DOI: 10.1107/S2414314622000839/tk4073Isup3.cml

CCDC reference: 2129837

Additional supporting information:  crystallographic information; 3D view; checkCIF report

## Figures and Tables

**Table 1:** Experimental details

Crystal data	
Chemical formula	C_20_H_24_BNO_2_
*M*_r_	321.23
Crystal system, space group	Monoclinic, *C*2/*c*
Temperature (K)	93
*a*, *b*, *c* (Å)	32.071 (11), 6.011 (2), 22.219 (8)
β (°)	122.590 (4)
*V* (Å^3^)	3609 (2)
*Z*	8
Radiation type	Mo *K*α
μ (mm^−1^)	0.07
Crystal size (mm)	0.13 × 0.11 × 0.05

Data collection	
Diffractometer	Rigaku Saturn724+
Absorption correction	Numerical (*NUMABS*; Rigaku, 1999 ▸)
*T*_min_, *T*_max_	0.991, 0.996
No. of measured, independent and observed [*F* ^2^ > 2.0σ(*F* ^2^)] reflections	13892, 3869, 3041
*R*_int_	0.084
(sin θ/λ)_max_ (Å^−1^)	0.639

Refinement	
*R*[*F* ^2^ > 2σ(*F* ^2^)], *wR*(*F* ^2^), *S*	0.052, 0.147, 1.09
No. of reflections	3869
No. of parameters	217
H-atom treatment	H-atom parameters constrained
Δρ_max_, Δρ_min_ (e Å^−3^)	0.25, −0.27

Computer programs: *CrystalClear* (Rigaku, 2008 ▸), *CrystalStructure* (Rigaku, 2019 ▸), *SHELXS* and *SHELXL* (Sheldrick, 2008 ▸), *ORTEP-3 for Windows* (Farrugia, 2012 ▸) and *publCIF* (Westrip, 2010 ▸).

## full crystallographic data

### Crystal data

**Table:**

C_20_H_24_BNO_2_	*F*(000) = 1376.00
*M**_r_* = 321.23	*D*_x_ = 1.182 Mg m^−^^3^
Monoclinic, *C*2/*c*	Mo *K*α radiation, λ = 0.71075 Å
*a* = 32.071 (11) Å	Cell parameters from 5341 reflections
*b* = 6.011 (2) Å	θ = 1.5–31.1°
*c* = 22.219 (8) Å	µ = 0.07 mm^−^^1^
β = 122.590 (4)°	*T* = 93 K
*V* = 3609 (2) Å^3^	Prism, colourless
*Z* = 8	0.13 × 0.11 × 0.05 mm

### Data collection

**Table:**

Rigaku Saturn724+ diffractometer	3041 reflections with *F*^2^ > 2.0σ(*F*^2^)
Detector resolution: 7.111 pixels mm^-1^	*R*_int_ = 0.084
ω scans	θ_max_ = 27.0°, θ_min_ = 1.5°
Absorption correction: numerical (NUMABS; Rigaku, 1999)	*h* = −33→40
*T*_min_ = 0.991, *T*_max_ = 0.996	*k* = −7→7
13892 measured reflections	*l* = −28→26
3869 independent reflections	

### Refinement

**Table:**

Refinement on *F*^2^	Secondary atom site location: difference Fourier map
*R*[*F*^2^ > 2σ(*F*^2^)] = 0.052	Hydrogen site location: inferred from neighbouring sites
*wR*(*F*^2^) = 0.147	H-atom parameters constrained
*S* = 1.09	*w* = 1/[σ^2^(*F*_o_^2^) + (0.0758*P*)^2^ + 0.8943*P*] where *P* = (*F*_o_^2^ + 2*F*_c_^2^)/3
3869 reflections	(Δ/σ)_max_ < 0.001
217 parameters	Δρ_max_ = 0.25 e Å^−^^3^
0 restraints	Δρ_min_ = −0.27 e Å^−^^3^
Primary atom site location: structure-invariant direct methods	

### Special details

**Table:**

Geometry. All esds (except the esd in the dihedral angle between two l.s. planes) are estimated using the full covariance matrix. The cell esds are taken into account individually in the estimation of esds in distances, angles and torsion angles; correlations between esds in cell parameters are only used when they are defined by crystal symmetry. An approximate (isotropic) treatment of cell esds is used for estimating esds involving l.s. planes.
Refinement. Refinement was performed using all reflections. The weighted R-factor (wR) and goodness of fit (S) are based on F^2^. R-factor (gt) are based on F. The threshold expression of F^2^ > 2.0 sigma(F^2^) is used only for calculating R-factor (gt).The C-bound H atoms were placed at ideal positions and were refined as riding on their parent C atoms. The *U*_iso_(H) values were set at 1.2*U*_eq_(C_sp2_) and 1.5 *U*_eq_(C_sp3_).

### Fractional atomic coordinates and isotropic or equivalent isotropic displacement parameters (Å^2^)

**Table:**

	*x*	*y*	*z*	*U*_iso_*/*U*_eq_	
O1	0.34742 (4)	0.98270 (18)	0.51068 (5)	0.0221 (3)	
O2	0.41736 (4)	0.82168 (18)	0.60407 (5)	0.0218 (3)	
N1	0.36028 (5)	0.6152 (2)	0.35305 (7)	0.0199 (3)	
C1	0.32337 (5)	0.6584 (3)	0.28043 (8)	0.0190 (3)	
C2	0.29881 (6)	0.8631 (3)	0.25973 (8)	0.0218 (3)	
H2	0.3090	0.9801	0.2936	0.026*	
C3	0.25961 (6)	0.8955 (3)	0.18984 (8)	0.0242 (4)	
H3	0.2427	1.0342	0.1764	0.029*	
C4	0.24475 (6)	0.7281 (3)	0.13936 (8)	0.0242 (4)	
H4	0.2173	0.7497	0.0919	0.029*	
C5	0.27059 (6)	0.5283 (3)	0.15912 (8)	0.0239 (4)	
H5	0.2613	0.4146	0.1244	0.029*	
C6	0.30963 (6)	0.4925 (3)	0.22861 (8)	0.0217 (3)	
H6	0.3271	0.3555	0.2412	0.026*	
C7	0.39973 (5)	0.4638 (2)	0.36945 (8)	0.0186 (3)	
C8	0.40344 (6)	0.2606 (3)	0.40195 (8)	0.0220 (3)	
H8	0.3798	0.2207	0.4133	0.026*	
C9	0.44182 (6)	0.1166 (3)	0.41769 (8)	0.0237 (3)	
H9	0.4446	−0.0221	0.4401	0.028*	
C10	0.47629 (6)	0.1748 (3)	0.40080 (8)	0.0246 (4)	
H10	0.5025	0.0757	0.4115	0.030*	
C11	0.47247 (6)	0.3772 (3)	0.36832 (8)	0.0248 (4)	
H11	0.4960	0.4166	0.3567	0.030*	
C12	0.43426 (6)	0.5225 (3)	0.35273 (8)	0.0226 (3)	
H12	0.4317	0.6617	0.3307	0.027*	
C13	0.35865 (6)	0.7131 (2)	0.40803 (8)	0.0194 (3)	
H13	0.3305	0.8029	0.3942	0.023*	
C14	0.39193 (6)	0.6968 (3)	0.47847 (8)	0.0206 (3)	
H14	0.4197	0.6011	0.4957	0.025*	
C15	0.34973 (6)	1.0522 (3)	0.57530 (8)	0.0210 (3)	
C16	0.40496 (6)	1.0063 (3)	0.63455 (8)	0.0226 (3)	
C17	0.31353 (6)	0.9064 (3)	0.58176 (10)	0.0296 (4)	
H17A	0.3140	0.9481	0.6247	0.036*	
H17B	0.2801	0.9272	0.5396	0.036*	
H17C	0.3232	0.7500	0.5851	0.036*	
C18	0.33465 (6)	1.2945 (3)	0.56815 (9)	0.0261 (4)	
H18A	0.3361	1.3421	0.6115	0.031*	
H18B	0.3573	1.3857	0.5616	0.031*	
H18C	0.3008	1.3124	0.5267	0.031*	
C19	0.41396 (7)	0.9327 (3)	0.70589 (9)	0.0344 (4)	
H19A	0.4058	1.0548	0.7270	0.041*	
H19B	0.3931	0.8038	0.6988	0.041*	
H19C	0.4488	0.8920	0.7380	0.041*	
C20	0.43914 (6)	1.1987 (3)	0.64438 (10)	0.0311 (4)	
H20A	0.4321	1.3267	0.6648	0.037*	
H20B	0.4737	1.1530	0.6767	0.037*	
H20C	0.4336	1.2400	0.5980	0.037*	
B1	0.38529 (6)	0.8323 (3)	0.53116 (9)	0.0188 (4)	

### Atomic displacement parameters (Å^2^)

**Table:**

	*U*^11^	*U*^22^	*U*^33^	*U*^12^	*U*^13^	*U*^23^
O1	0.0242 (6)	0.0246 (6)	0.0168 (6)	0.0045 (4)	0.0105 (5)	−0.0009 (4)
O2	0.0246 (6)	0.0227 (6)	0.0180 (6)	0.0047 (4)	0.0114 (5)	−0.0001 (4)
N1	0.0220 (7)	0.0207 (6)	0.0176 (6)	0.0024 (5)	0.0110 (6)	−0.0006 (5)
C1	0.0176 (7)	0.0236 (8)	0.0171 (7)	−0.0014 (6)	0.0101 (6)	0.0001 (6)
C2	0.0258 (8)	0.0213 (8)	0.0214 (8)	−0.0001 (6)	0.0149 (7)	−0.0009 (6)
C3	0.0248 (8)	0.0283 (8)	0.0242 (8)	0.0046 (6)	0.0164 (7)	0.0051 (6)
C4	0.0211 (8)	0.0338 (9)	0.0172 (8)	−0.0005 (6)	0.0101 (7)	0.0024 (6)
C5	0.0247 (8)	0.0298 (8)	0.0201 (8)	−0.0048 (6)	0.0139 (7)	−0.0042 (6)
C6	0.0239 (8)	0.0219 (8)	0.0233 (8)	0.0005 (6)	0.0154 (7)	−0.0004 (6)
C7	0.0178 (7)	0.0201 (7)	0.0159 (7)	0.0012 (6)	0.0077 (6)	−0.0024 (6)
C8	0.0215 (8)	0.0241 (8)	0.0224 (8)	−0.0025 (6)	0.0132 (7)	−0.0023 (6)
C9	0.0256 (8)	0.0209 (8)	0.0231 (8)	0.0005 (6)	0.0121 (7)	−0.0002 (6)
C10	0.0215 (8)	0.0267 (8)	0.0226 (8)	0.0024 (6)	0.0099 (7)	−0.0038 (6)
C11	0.0216 (8)	0.0312 (9)	0.0244 (8)	−0.0018 (6)	0.0143 (7)	−0.0035 (7)
C12	0.0253 (8)	0.0236 (8)	0.0206 (8)	−0.0007 (6)	0.0135 (7)	−0.0002 (6)
C13	0.0210 (8)	0.0183 (7)	0.0241 (8)	−0.0015 (6)	0.0155 (7)	−0.0021 (6)
C14	0.0202 (8)	0.0212 (7)	0.0216 (8)	0.0012 (6)	0.0121 (7)	−0.0011 (6)
C15	0.0252 (8)	0.0207 (7)	0.0203 (8)	0.0021 (6)	0.0143 (7)	−0.0009 (6)
C16	0.0272 (8)	0.0227 (8)	0.0195 (8)	0.0014 (6)	0.0136 (7)	−0.0011 (6)
C17	0.0329 (9)	0.0244 (8)	0.0404 (10)	−0.0022 (7)	0.0256 (8)	−0.0032 (7)
C18	0.0313 (9)	0.0214 (8)	0.0286 (9)	0.0041 (6)	0.0182 (8)	0.0009 (6)
C19	0.0430 (10)	0.0406 (10)	0.0193 (8)	0.0060 (8)	0.0166 (8)	0.0008 (7)
C20	0.0280 (9)	0.0308 (9)	0.0300 (9)	−0.0045 (7)	0.0127 (8)	−0.0091 (7)
B1	0.0193 (8)	0.0187 (8)	0.0203 (9)	−0.0020 (6)	0.0118 (7)	−0.0007 (6)

### Geometric parameters (Å, º)

**Table:**

O1—B1	1.380 (2)	C10—H10	0.9500
O1—C15	1.4585 (18)	C11—C12	1.388 (2)
O2—B1	1.375 (2)	C11—H11	0.9500
O2—C16	1.4623 (18)	C12—H12	0.9500
N1—C13	1.3824 (19)	C13—C14	1.341 (2)
N1—C1	1.419 (2)	C13—H13	0.9500
N1—C7	1.4369 (19)	C14—B1	1.532 (2)
C1—C2	1.398 (2)	C14—H14	0.9500
C1—C6	1.403 (2)	C15—C18	1.516 (2)
C2—C3	1.388 (2)	C15—C17	1.522 (2)
C2—H2	0.9500	C15—C16	1.560 (2)
C3—C4	1.386 (2)	C16—C19	1.516 (2)
C3—H3	0.9500	C16—C20	1.526 (2)
C4—C5	1.390 (2)	C17—H17A	0.9800
C4—H4	0.9500	C17—H17B	0.9800
C5—C6	1.384 (2)	C17—H17C	0.9800
C5—H5	0.9500	C18—H18A	0.9800
C6—H6	0.9500	C18—H18B	0.9800
C7—C12	1.390 (2)	C18—H18C	0.9800
C7—C8	1.391 (2)	C19—H19A	0.9800
C8—C9	1.387 (2)	C19—H19B	0.9800
C8—H8	0.9500	C19—H19C	0.9800
C9—C10	1.390 (2)	C20—H20A	0.9800
C9—H9	0.9500	C20—H20B	0.9800
C10—C11	1.386 (2)	C20—H20C	0.9800
			
B1—O1—C15	107.10 (11)	N1—C13—H13	116.0
B1—O2—C16	107.02 (12)	C13—C14—B1	120.13 (14)
C13—N1—C1	121.41 (13)	C13—C14—H14	119.9
C13—N1—C7	119.53 (13)	B1—C14—H14	119.9
C1—N1—C7	119.05 (12)	O1—C15—C18	109.14 (12)
C2—C1—C6	118.90 (14)	O1—C15—C17	106.93 (13)
C2—C1—N1	120.80 (14)	C18—C15—C17	110.29 (13)
C6—C1—N1	120.25 (14)	O1—C15—C16	102.26 (12)
C3—C2—C1	120.12 (15)	C18—C15—C16	114.30 (13)
C3—C2—H2	119.9	C17—C15—C16	113.29 (13)
C1—C2—H2	119.9	O2—C16—C19	108.47 (13)
C4—C3—C2	120.84 (15)	O2—C16—C20	106.72 (13)
C4—C3—H3	119.6	C19—C16—C20	110.72 (14)
C2—C3—H3	119.6	O2—C16—C15	102.24 (12)
C3—C4—C5	119.03 (15)	C19—C16—C15	115.10 (14)
C3—C4—H4	120.5	C20—C16—C15	112.83 (13)
C5—C4—H4	120.5	C15—C17—H17A	109.5
C6—C5—C4	120.93 (15)	C15—C17—H17B	109.5
C6—C5—H5	119.5	H17A—C17—H17B	109.5
C4—C5—H5	119.5	C15—C17—H17C	109.5
C5—C6—C1	120.06 (15)	H17A—C17—H17C	109.5
C5—C6—H6	120.0	H17B—C17—H17C	109.5
C1—C6—H6	120.0	C15—C18—H18A	109.5
C12—C7—C8	120.28 (14)	C15—C18—H18B	109.5
C12—C7—N1	119.38 (14)	H18A—C18—H18B	109.5
C8—C7—N1	120.34 (13)	C15—C18—H18C	109.5
C9—C8—C7	119.68 (14)	H18A—C18—H18C	109.5
C9—C8—H8	120.2	H18B—C18—H18C	109.5
C7—C8—H8	120.2	C16—C19—H19A	109.5
C8—C9—C10	120.11 (15)	C16—C19—H19B	109.5
C8—C9—H9	119.9	H19A—C19—H19B	109.5
C10—C9—H9	119.9	C16—C19—H19C	109.5
C11—C10—C9	120.09 (15)	H19A—C19—H19C	109.5
C11—C10—H10	120.0	H19B—C19—H19C	109.5
C9—C10—H10	120.0	C16—C20—H20A	109.5
C10—C11—C12	120.06 (15)	C16—C20—H20B	109.5
C10—C11—H11	120.0	H20A—C20—H20B	109.5
C12—C11—H11	120.0	C16—C20—H20C	109.5
C11—C12—C7	119.77 (15)	H20A—C20—H20C	109.5
C11—C12—H12	120.1	H20B—C20—H20C	109.5
C7—C12—H12	120.1	O2—B1—O1	112.71 (13)
C14—C13—N1	127.95 (14)	O2—B1—C14	123.48 (14)
C14—C13—H13	116.0	O1—B1—C14	123.79 (14)
			
C13—N1—C1—C2	30.2 (2)	C1—N1—C13—C14	−176.36 (15)
C7—N1—C1—C2	−150.72 (14)	C7—N1—C13—C14	4.5 (2)
C13—N1—C1—C6	−147.37 (14)	N1—C13—C14—B1	175.57 (14)
C7—N1—C1—C6	31.7 (2)	B1—O1—C15—C18	145.27 (13)
C6—C1—C2—C3	3.7 (2)	B1—O1—C15—C17	−95.43 (14)
N1—C1—C2—C3	−173.86 (13)	B1—O1—C15—C16	23.86 (15)
C1—C2—C3—C4	−1.1 (2)	B1—O2—C16—C19	146.11 (14)
C2—C3—C4—C5	−1.8 (2)	B1—O2—C16—C20	−94.56 (14)
C3—C4—C5—C6	2.1 (2)	B1—O2—C16—C15	24.11 (14)
C4—C5—C6—C1	0.6 (2)	O1—C15—C16—O2	−28.89 (14)
C2—C1—C6—C5	−3.4 (2)	C18—C15—C16—O2	−146.69 (13)
N1—C1—C6—C5	174.15 (13)	C17—C15—C16—O2	85.81 (15)
C13—N1—C7—C12	−113.40 (16)	O1—C15—C16—C19	−146.25 (14)
C1—N1—C7—C12	67.48 (19)	C18—C15—C16—C19	95.96 (17)
C13—N1—C7—C8	66.41 (19)	C17—C15—C16—C19	−31.54 (19)
C1—N1—C7—C8	−112.71 (16)	O1—C15—C16—C20	85.36 (15)
C12—C7—C8—C9	0.0 (2)	C18—C15—C16—C20	−32.44 (18)
N1—C7—C8—C9	−179.76 (14)	C17—C15—C16—C20	−159.93 (13)
C7—C8—C9—C10	−0.2 (2)	C16—O2—B1—O1	−10.22 (17)
C8—C9—C10—C11	0.2 (2)	C16—O2—B1—C14	168.01 (14)
C9—C10—C11—C12	0.1 (2)	C15—O1—B1—O2	−9.76 (17)
C10—C11—C12—C7	−0.3 (2)	C15—O1—B1—C14	172.01 (14)
C8—C7—C12—C11	0.3 (2)	C13—C14—B1—O2	178.88 (14)
N1—C7—C12—C11	−179.94 (13)	C13—C14—B1—O1	−3.1 (2)
